# State of the Art of Different Zirconia Materials and Their Indications According to Evidence-Based Clinical Performance: A Narrative Review

**DOI:** 10.3390/dj11010018

**Published:** 2023-01-04

**Authors:** Alexia María Arellano Moncayo, Lissethe Peñate, María Arregui, Luis Giner-Tarrida, Rosario Cedeño

**Affiliations:** 1 Independent Researcher, Doha 00000, Qatar; 2 Department of Restorative Dentistry, Faculty of Dentistry, Universitat Internacional de Catalunya, 08195 Sant Cugat del Valles, Spain; 3 Department of Dentistry, Faculty of Dentistry, Universitat Internacional de Catalunya, 08195 Sant Cugat del Valles, Spain

**Keywords:** zirconia materials, ZrO_2_ generations, ZrO_2_ indications

## Abstract

The aim of this study was to perform a narrative review to identify the modifications applied to the chemical structure of third- and fourth-generation zirconia ceramics and to determine the influence of these changes on the mechanical and optical properties. A bibliographical search using relevant keywords was conducted in the PubMed^®^ and EBSCO databases. The abstracts and full texts of the resulting articles were reviewed for final inclusion. Fifty-four articles were included in this review. The analyzed topics were: (1) the composition of first- and second-generation zirconia materials (Y-TZP), (2) the behavior of the studied generations in relation to mechanical and optical properties, and (3) the modifications that were carried out on third-generation (5Y-TZP) and fourth-generation (4Y-TZP) zirconia materials. However, studies focusing on these specific characteristics in third- and fourth-generation zirconia materials are scarce. The review shows that there is a lack of sufficient knowledge about the chemical modifications of zirconia in the new generations.

## 1. Introduction

The natural appearance results in fixed dental prosthesis (FDPs) treatments have been guided by the evolution of dental ceramics; zirconium dioxide (ZrO_2_) is part of this evolution [1,2,3,4,5,6]. 

The polycrystalline structure of zirconium dioxide (ZrO_2_) is arranged on cells (mesh) shaped in three different phases: cubic, tetragonal, and monoclinic. They transform from one phase into another, induced by a combination of temperature, humidity, and stress stimulus [7,8,9,10,11,12,13,14,15,16].

Three mol% yttria-stabilized tetragonal zirconia polycrystalline (3Y-TZP) is a ceramic system that exhibits high strength, fracture toughness, hardness, wear resistance, good frictional and non-magnetic behavior, electrical insulation, low thermal conductivity, corrosion resistance in acids and alkalis, modulus of elasticity similar to steel, and coefficient of thermal expansion similar to iron. However, poor translucency has been one of the characteristics to improve in this material [17,18,19,20,21,22,23,24,25,26,27,28,29]. 

Yttria-stabilized tetragonal zirconia polycrystalline is termed Y-TZP. Zirconia-based ceramics used for biomedical purposes typically exist as a metastable tetragonal partially stabilized zirconia (PSZ), which means that trapped energy still exists within the material, preventing the system from transforming into the monoclinic phase at room temperatures. However, 3 mol% yttria-stabilized tetragonal zirconia polycrystalline (3Y-TZP) suffers a phase transformation when mechanical or physical stress is applied. It has been observed that fatigue creates micro-cracks in the structure of zirconia materials. When a crack propagates in 3Y-TZP (that has these metastable tetragonal particles), a stress-induced transformation occurs at the end of the crack and only the particles near this transform from the tetragonal into the monoclinic phase; this process is called transformation toughening (TT) [6,10]. This transformation toughens the material in the following two different ways: the energy needed for fracture is first dissipated during transformation and second through residual compressive stress [4,7,11,21,25].

On the other hand, 3Y-TZP under oral conditions has shown an accelerated aging, affecting a long lasting prosthesis. Scientists who have investigated the biomaterial behavior have named this phenomenon as “low temperature degradation (LTD)”, where the tetragonal zirconia (t-ZrO_2_) phase spontaneously transforms to the monoclinic (m-ZrO_2_) phase as a response to the variation of temperature in the mouth and the presence of humidity (hydrothermal aging), regardless of any mechanical stress. The consequences of hydrothermal aging are surface roughening due to loss of crystals, enhanced wear rates, detrimental strength, and fracture toughness, followed by catastrophic failures [4,8,11,12,16].

To maintain stability of the crystalline form at room temperature yttria, magnesia, and other oxides have been added to zirconia materials. Güth et al. [6] described four generations of zirconia material based on its chemical structure: first-generation 3 mol% Y_2_O_3_ 0.25% Al_2_O_3_ (3Y-TZP); second-generation 3 mol% Y_2_O_3_ 0.05% Al_2_O_3_; third-generation 5 mol% Y_2_O_3_ 0.05% Al_2_O_3_ 53% cubic structure (5Y-TZP); fourth-generation 4 mol% Y_2_O_3_ 0.05% Al_2_O_3_ (4Y-TZP). 

In 2015, a new ceramic system was introduced to the market: the third-generation 5-mol% yttria-stabilized tetragonal zirconia polycrystal (5Y-TZP). Increasing the yttria content (Y₃O₂) to 5 mol% was a modification that offered enhancement in the translucency of zirconia. The result is a fully stabilized zirconia with a stable cubic–tetragonal microstructure. The cubic phase reaches approximately 50% of the structure. The size and number of the crystals, which are larger than the 3Y-TZP, favor the light transmission, reducing the refraction effect and giving better translucency. On the other hand, an increase in the number of cubic crystals affects the crack propagation pattern, reducing the flexural strength and the fracture toughness of the material [1,6,10,12,30]. 

It is relevant to recall the findings attained by Zhang et al. [13] in 2020 that describe 3Y-TZP ceramics. These ceramics allow less light transmission because of the large refraction of light in two different directions (optically anisotropic)—an effect of the tetragonal phase—causing light diffusion and light deflection at the grain boundaries that enhance the translucency of zirconia ceramics [12,17,31,32,33,34].

The mechanism for increasing yttria oxide as a strategy to reach a higher translucency in the zirconia materials involves better aging stability but lower flexural strength and fracture toughness, mainly due to the change in the phase composition [35,36,37,38,39]. Compared with 3Y-TZP that consists of ~80 wt% tetragonal phase and ~20 wt% cubic phase, PSZ stabilized with 4–6 mol% yttria used for dental purposes contains a 40–70 wt% cubic phase influenced by the yttria stabilizer concentration and the sintering temperature. Increasing the cubic phase in the zirconia structure transforms the composition into a nonbirefringent (refraction in two directions) and non-transformable (loss of grains and aging) under humidity and stress conditions [13,14,15,17,18,19].

In 2017, manufacturing companies launched the fourth-generation 4-mol% yttria-stabilized tetragonal zirconia polycrystals (4Y-TZP) of zirconia. Compared with the third-generation, the yttria content was reduced to 4-mol%, which led to an enhancement of the flexural strength and fracture toughness, with a combined reduction in translucency [6,10,14,31]. Comparing the strength, toughness, and translucency of zirconia restorations allows clinicians and dental lab technicians to make assertive decisions based on the evidence of prosthetic restoration on one or more than three units. For this reason, the research question sought to identify the expectancy of an enhancement of the different properties when comparing the third- and fourth-generation of zirconia materials.

The aim of this study was to retrieve the papers that analyze the modifications performed in the chemical structure of third- and fourth-generation zirconia ceramics in order to determine how these structural changes have influenced the strength, toughness, and translucency of these materials. 

## 2. Materials and Methods

### 2.1. Study Selection

This study consisted of an extensive search of the literature available on dental materials worldwide. The search was limited exclusively to papers in English, between 2015 and 2022, that feature on the two main search engines (PubMed^®^ and EBSCO) and that are published in of the most influential indexed journals in the materials and dental field. A first search was performed in October 2021 and a second search was conducted in July of 2022. 

### 2.2. Inclusion Criteria

Clinical trials (randomized controlled trials), meta-analysis, systematic reviews, in vitro studies, and literature reviews were included in the present study. The following characteristics were taken into account: zirconia materials, translucent zirconia, cubic zirconia, ultra-translucent zirconia, mechanical properties, chemical structure, and load bearing of translucent zirconia.

### 2.3. Exclusion Criteria

Books and documents, all papers in a foreign language (not in the English language), and zirconia materials investigated in other applications different from fixed prosthodontics were excluded.

### 2.4. Study Quality Assessment

The search strategy was designed and set up by one reviewer (A.A.), who also performed the electronic search. All the studies that fulfilled the inclusion criteria were included in this review. The title and abstracts of all articles identified by the electronic search were read and assessed by one of the authors (A.A.). A shortlist of studies was then compiled and subjected to full text analysis and data extraction by the contributing authors. A manual search of the analyzed articles was also carried out. The methodological quality of all selected full-text articles was assessed using the guidelines given by PRISMA and CONSORT [40].

First, the authors analyzed those papers that described zirconium oxide as a pure element in nature and its modifications, including its chemical structure, phase transformation, and low-temperature degradation. Second, the authors classified those papers that reported data on third- and fourth-generation zirconia materials. 

## 3. Results

**Figure 1:**
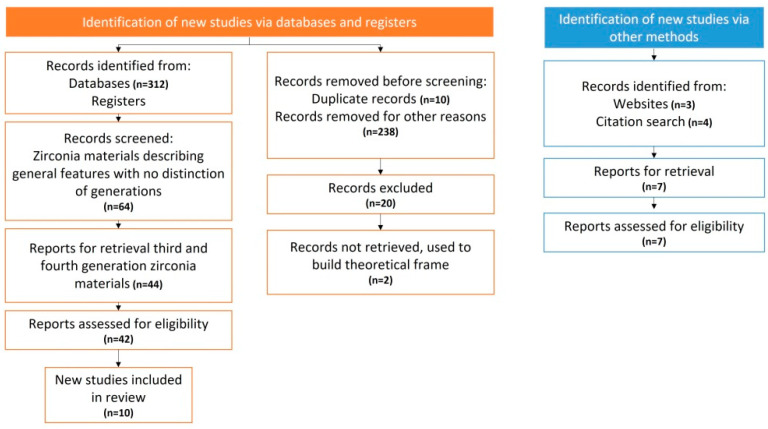
Flow chart of the literature search.

The original search strategy—based on keywords mentioned—resulted in 322 papers. However, the total number of papers that met the inclusion criteria for the review was 64, whereby 45.6% addressed first- and second-generation zirconia materials and 54.3% studied third- and fourth-generation zirconia materials. A total of 64 articles was reviewed, of which 20 were discarded and 44 related to third- and fourth-generation zirconia were used, with a further 10 articles added to address generalities of the review. The flow chart of the obtained results of the literature search is given in Figure 1.

This study aimed to understand the chemical composition of zirconia ceramics and to determine which modifications have been carried out. The first selection of papers allowed the authors to categorize the composition of first- and second-generation zirconia materials (Y-TZP) and to identify the behavior of the studied generations of zirconia materials regarding abrasiveness and wear, marginal accuracy, and cementation. The second selection permitted to establish the modifications that were carried out in third-generation (5Y-TZP) and fourth-generation (4Y-TZP) zirconia materials, allowing to understand how these modifications have influenced the flexural strength, fracture toughness, and translucency of the available materials. Studies that addressed these specific characteristics in third- and fourth-generation are scarce.

All selected papers are distributed according to the information they offer regarding the composition and features of zirconia materials. A summary of the in vitro studies and the observational study articles reviewed in this paper can be found in Table 1.

## 4. Discussion

Zirconia materials have evolved into several formulations, depending on powder composition, sintering additives, heat treatment, and other processing factors.

### 4.1. Third-Generation Y-TZP: Increasing Yttria Content

For Y-TZP, the translucency properties are tailored by its cubic content, which can be controlled by both the yttria content and the sintering temperature. Increasing the yttria content gives way to third-generation zirconia materials (5Y-TZP or 5Y-0.05Al) 5 mol% Y_2_O_3_ 0.05% Al_2_O_3_. Due to 53% of its structure being found in cubic polycrystals, it received the name of cubic zirconia or the so-called fully stabilized zirconia (FSZ) [3,4,5,6,7,8,9,10,11,12,15,17,18,19,35,36].

In general, a higher yttria content and sintering temperature will have a greater cubic content and better translucency. Nevertheless, this also triggers a lower strength and toughness [1,5,10,12]. The structure allowed (5Y-0.05Al) to have a much higher translucency than other (3Y-TZP) ceramic systems, which contained about 90% birefringent tetragonal zirconia. Cubic zirconia is optically isotropic, without light scattering at the grain boundaries [29,34,43,47,48,49]. Moreover, since cubic zirconia is a stable phase and the yttria content in the residual tetragonal zirconia of (5Al-0.05Al) was high (about 3.9 mol%), 5Y-0.05Al was resistant to hydrothermal aging [16]. However, the mechanical properties of (5Y-0.05Al) are a crucial drawback. Cubic zirconia is brittle and the tetragonal zirconia with a higher yttria content has a lower ability of transformation toughening. Therefore, (5Y-0.05Al) had lower fracture toughness. The low toughness, combined with the larger grain size of (5Y0.05Al), also resulted in a much lower strength [5,10,12,15,17]. Third-generation zirconia (5Y-TZP) exhibits 35–40% translucency and 500 MPa BFS, which does not fulfill the mechanical requirements for multiple-unit fixed dental prosthesis [9,15]. Increasing the content of yttrium oxide in an attempt to improve the optical properties can reduce the strength and toughness after aging of the ceramic [9,15,17].

### 4.2. Fourth-Generation Y-TZP: Decreasing Yttria Content

Fourth-generation zirconia materials are created to enhance strength and toughness, but again translucency and light reflection are diminished. The (4Y-TZP) 4 mol% Y_2_O_3_ 0.05% Al_2_O_3_ exhibits 30% translucency, 900 MPa BFS, and better aging resistance [9,15,18,19].

Currently, three zirconia grades based on the percentage of yttria content are available for monolithic dental restorations, namely 3Y-, 4Y-, and 5Y-PSZ (mol% yttria partially stabilized zirconia). These three grades have a broad and inversely proportional balance between translucency and strength and, therefore, offer different clinical indications [6,18,20,21,22].

Zhang investigated the remaining tetragonal phase and its role in 4Y- and 5Y-PSZ (mol% yttria partially stabilized zirconia). Both materials had similar basic properties. However, 5Y-PSZ (mol% yttria partially stabilized zirconia) had a variation on the microstructure. When 5Y-PSZ was processed from an yttria co-precipitated powder, in which the 5 mol% Y_2_O_3_ stabilizer was already homogeneously distributed inside, the zirconia starting powder had a significantly higher translucency, biaxial strength, and aging stability, demonstrating that the cubic content and the microstructure of the remaining tetragonal grains had considerable influence on the properties of 4Y- and 5Y-PSZ (mol% yttria partially stabilized zirconia) [13,23,24,25,26,27].

The literature supports the findings regarding the difference in the properties between third- and fourth-generation zirconia materials. The third-generation shows better optical properties than the fourth-generation zirconia materials, but the fourth-generation zirconia exhibits better strength. 

The abrasiveness and wear are closely related to grain size; thus, third- and fourth-generation zirconia show a similar grade of wear that is very close to enamel wear [4,7,12,15,23]. Regarding surface treatment and cementation, third- and fourth-generation zirconia lack adhesiveness and need to undergo the same surface treatments to improve the surface roughness [25,26,27,28,29,33,50,51]. Alammar et al. [52], in 2022, conducted a systematic review on the bonding of high translucency zirconia and concluded that the bonding protocols already applied on conventional zirconia (particle abrasion treatment, MDP primers, and resin cements) provide the best results also on this type of zirconia, as they provide a long-term adhesive bond without compromising the physical properties.

Most of the studies that analyze the ageing of zirconia ceramics focus on the study of the influence of changes generated by hydrothermal conditions in the oral environment. These changes are influenced by the phase transformation of the zirconia and at the same time the affectation caused by crack propagation in the fracture, which is studied by using compressive stress. The aging resistance is higher in third- than fourth-generation zirconia due to the cubic phase percentage [4,7,12,17,32,38,42]. No differences were found in the marginal accuracy and internal fit of zirconia materials in the third and fourth generation [24,30,46]. Nevertheless, in recent years, especially after the emergence of translucent zirconia, there has been an increase in research on the cyclic fatigue of these materials to fill the gap in the literature on the dynamic ageing of these materials. As commented by Baldi et al. [53] in 2022, it is observed that there is a lower strength and marginal sealing of high translucent zirconia compared with zirconia-reinforced lithium silicate under cyclic fatigue, so there is still a need for further research.

The manufacturing and processing techniques affect the optical properties of zirconia-based restorations [3,9,12,17,30,45]. Restorations manufactured with the full-contour technique using 4YT ZP-MT were the darkest and most translucent. The 3YTZP-LT produces the lightest and least-translucent restorations [3,5,10,12,14,30,31,32,36,43,45,46,54]. UV aging caused zirconia-based restorations to be darker and more yellow, red, saturated, and opaque [17,31,32,33,34].

Despite the previous efforts that have been devoted to the study of this material, there is evidently a lack of a comprehensive understanding of the chemical structure. An absence of unified terminology and several strategies adopted by manufacturing companies to improve the characteristics of this ceramic system originate a gap in the knowledge that allows mistakes when clinicians and lab technicians are using Y-TZP as a ceramic material. A limitation of this review of the literature is that the behavior of new generations of zirconia under cyclic fatigue has not been analyzed. Future research needs to analyze the cyclic fatigue and the clinical behavior of this material.

## 5. Conclusions

Zirconia ceramics are widely used in the biomedical field. Regarding the physicochemical features and optical and mechanical properties, the expectancy of an enhancement of the mechanical properties with a combined reduction in its light optical properties when comparing the third- and fourth-generation zirconia was confirmed. The fourth-generation zirconia material 4Y-TZP shows better mechanical properties but less percentage of translucency. Clinicians should be careful when zirconia material is their choice when performing fixed restorations. Translucency is not always an advantage; if discolored stumps were the base of their restoration, translucent zirconia materials would become disadvantageous.

## Figures and Tables

**Table 1:** Summary of clinical and in vitro studies analyzed.

Year of Publishing (Study Design)	Authors (Country)	Aim	Sample Size	Material	Zirconia Type	Results	Conclusions
2020 (in vitro)	Zucuni et al. [32] (Brazil)	Evaluate the effect of distinct surface treatments (grinding, polishing, and glaze) of fully stabilized zirconia (FSZ).	Disc of FSZ (n = 15). Size: 0.8 mm in thickness and 10 mm in diameter.	ZirCAD MT Multi (Ivoclar Vivadent, Schaan, Liechtenstein)	**4Y-TZP**	Grinding generates scratches and defects on the material’s surface. Polishing and glaze show surface smoothening effects. None of the surface treatments affected the mechanical properties.	The surface treatments of the occlusal surface of zirconia restorations do not impair the fatigue behavior of the restorations. The ground FSZ surface should be polished or glazed for smoothening effect.
2020 (in vitro)	Moqbel et al. [15] (Germany)	Evaluate the influence of aging and surface treatment on surface roughness, biaxial flexural strength (BFS), and Vickers hardness (VHN) of translucent dental zirconia.	Eighty disc-shaped zirconia specimens. Size: 1.2 mm in thickness and 12 mm in diameter. Half were not aged and the other half were aged in autoclave for 20 hrs.	High translucent zirconia (Katana HT10, Kuraray, Tokyo, Japan)	**4Y-TZP**	Aging and particle air abrasion increased the BFS. The hardness was not influenced significantly by aging.	Particle air abrasion and aging demonstrated a significant transformation from tetragonal phase to-monoclinic phase, which led to significant increase of BFS.
2019 (in vitro)	Na- Kyoung Yu et al. [41] (Korea)	Evaluate the effect of two coloring liquids and the position of multi-layered zirconia on flexural strength.	Sixty multi-layered specimens were divided into incisal and cervical positions. Three subgroups (n = 10): non-shaded, acid-based coloring liquid, and aqueous coloring liquid.	3M Lava Esthetic (3M Deutschland GmbH, Germany)	**5Y-TZP**	The flexural strength of multi-layered zirconia was 400–500 MPa. There was no statistically significant difference among all groups.	The different coloring liquids did not affect the flexural strength of multi-layered zirconia of all positions
2019 (in vitro)	Kurtulmus-Yilmaz et al. [28] (Turkey)	Investigate the influence of surface treatments conducted in pre- sintering and post- sintering stages on flexural strength and optical properties of zirconia.	Zirconia blocks partially sintered were milled in different geometries for three tests. Flexural strength (n = 70): bar- shaped. Color evaluation (n = 70): square-shaped. Topographic analysis (n = 12): disc-shaped. Groups: pre-sintered, post-sintered, and control.	GC Initial zirconia disk (GC America Inc, EEUU)	**3Y-TZP**	Post-sintered groups exhibited higher values on mechanical properties.	Surface treatments performed at the post- sintering stage had a favorable effect on flexural strength. Surface treatments performed before sintering increased translucency and caused higher ΔE_00_ values.
2019 (in vitro)	Jansen et al. [9] (Germany)	Test and compare two high-speed sintering protocols and one conventional sintering protocol on the translucency, phase content, grain sizes, and flexural strength of three zirconia materials.	Total of 450 specimens of 3 zirconia materials (n = 150)	3Y-TZP: Ceramill (ZI) and Zolid (ZD). 4Y-TZP: Zolid (HT) Ceramill Motion 2 (Amann Girrbach AG)	**3Y-TZP** 20% translucency, 1200 MPa BFS. **3Y-TZP** reduced Al_2_O_3_ 25% translucency, 1000 MPa BFS. **4Y-TZP** 30% translucency, 900 MPa BFS.	**ZI**: sintering protocols did not affect the translucency of BFS. **ZD**: showed significantly lower translucency for high-speed protocols. **HT**: showed significantly lower translucency for high-speed protocols.	The biggest influence in translucency was exerted by the specimen thickness followed by the sintering protocol. No monoclinic phase was detected in any group. Grain size increased with increasing final sintering temperature. High-speed sintering increased BFS in ZD and HT. Translucency was decreased in ZD and HT.
2019 (in vitro)	Vieira Cardoso et al. [3] (Brazil)	Determine the effect of different sintering temperatures on the microstructure and optical properties of FSZ.	Bar-shaped FSZ specimens were divided into 2 groups (n = 15) according to final sintering temperatures (1450ºC and 1600ºC).	Fully stabilized zirconia blocks (Prettau^®^ Anterior, Zirkonzahn GmbH, Italy)	**5Y-TZP**	Reflectance and sum of light absorption-scattering values were significantly different. Translucency, opacity, and flexural strength showed no statistical differences.	Higher sintering temperatures increased the grain size but did not change the crystal phase concentration.
2019 (in vitro)	Elsayed et al. [36] (Germany)	Evaluate fracture strength of full-anatomical crowns manufactured from three different types of zirconia with three different contents of yttrium oxide.	Total of 48 specimens divided into three groups (n = 16): 3Y-TZP, 4Y-TZP, and 5Y-TZP.	Group A, DD Bio ZX2 (3Y-TZP) (Dental Direkt) Group B, DD cubeX2 HS (4Y-TZP) (Dental Direkt) Group C, DD cubeX2 (5Y-TZP) (Dental Direkt).	**3Y-TZP** **4Y-TZP** **5Y-TZP**	The highest fracture strength was shown in 3Y-TZP, the lowest in 5Y-TZP.	Increasing the content of yttrium oxide in an attempt to improve the optical properties can reduce the mechanical properties after aging of the ceramic.
2019 (in vitro)	Auzani et al. [31] (Brazil)	Evaluate the effect of shading procedures on fatigue performance and optical properties on 4Y-TZP.	Seventy-five discs of Y-TZP ceramic were divided into 5 groups (n = 15).	IPS e.max ZirCAD MT BL (Ivoclar Vivadent, Schaan, Liechtenstein)	**4Y-TZP**	Shading technique negatively affected the mechanical fatigue properties of 4Y-TZP.	The shading technique used for pigmentation affects translucency, opalescence, and fatigue properties of 4Y-TZP.
2019 (in vitro)	Zhang et al. [23] (Belgium)	Evaluate two-body wear of three zirconia ceramics stabilized with 3, 4, and 5% mol yttria and to compare their wear behavior with lithium-disilicate glass-ceramic.	Sixteen rectangular-shaped specimens from three grades of zirconia ceramics and a lithium-disilicate glass-ceramic.	Zpex (3Y-TZP) Zpex 4 (4Y-TZP) Zpex smile (5Y-TZP) (Tosoh, Tokyo, Japan). IPS e.max CAD blocks (IPS e.max CAD HT A2, Ivoclar Vivadent, Schaan, Liechtenstein)	**3Y-TZP** **4Y-TZP** **5Y-TZP**	The three zirconia ceramics showed a similar and limited amount of wear. The wear resistance was higher than lithium-disilicate.	The threshold in stress intensity for crack growth along with microstructural homogeneity and surface degradation are also key parameters that should be taken into account.
2019 (in vitro)	Kou et al. [42] (Sweden)	Analyze the effects of artificial aging on surface roughness, transparency, phase transformation, and BFS of different zirconia products.	Twenty discs. Size: 1.1 mm in thickness and 13 mm in diameter.	DD cubeX2 (Dental Direkt Gmbh, Spenge, Germany) Prettau Anterior (Zirkonzahn GmbH, Gais, Italy).	**4Y-TZP** **5Y-TZP**	DD cubeX2 had higher positive values on mechanical properties than Prettau Anterior both before and after artificial aging for 10 hrs.	Within the limitations of the present in vitro study, both DD cubeX2 and Prettau Anterior seem to be relatively aging resistant. However, a wider range of measured flexural strength indicated that Prettau Anterior probably is a less stable material than DD cubeX2, which also means that the flexural strength of DD cubeX2 could be more predictable.
2019 (in vitro)	Hayasi et al. [22] (Japan)	Clarify the influence of translucent tetragonal zirconia polycrystals on wear properties of esthetic dental materials.	Disc shaped. Size: 1.0 mm in thickness and 13 mm in diameter.	Zpex100 Zpex (Tosoh, Tokyo, Japan)	**5Y-TZP**	The wear volume of TZP was extremely small. Polished translucent TZP indicates that wear hardly occurs.	No visible wear was found on translucent TZP.
2019 (in vitro)	Elsaka et al. [43] (Egypt)	Evaluate the optical and mechanical properties of newly introduced monolithic multilayer zirconia with two types of monolithic zirconia.	Thirty bar-shaped specimens. Size: 18 mm × 4.0 mm × 3.0 mm	Ceramill Zolid FX Multilayer (CZF) (Amann Girrbach, Koblach, Austria) Prettau Anterior (PA) (Zirkonzahn GmbH, Bruneck, Italy) Zenostar Translucent (ZT) (Wieland Dental GmbH, Lindenstrabe, Germany)	CZF: **4Y-TZP** PA: **5Y-TZP** ZT: **4Y-TZP**	*Optical properties*: CZF revealed significantly higher translucency parameter (TP) and lower contrast ratio (CR) compared with PA and ZT monolithic zirconia. *Mechanical properties*: ZT showed significantly higher flexural strength and fracture toughness compared with CZF and PA. CZF revealed significant higher hardness values compared with PA and ZT. CZF and PA revealed higher brittleness index than ZT monolithic zirconia.	The optical and mechanical properties of the tested monolithic zirconia are material dependent. Fully stabilized monolithic zirconia materials (CZF and PA) are relatively more translucent than partially stabilized zirconia (ZT).
2018 (in vitro)	Ebeid et al. [44] (Germany)	Evaluate the effect of zirconia surface treatment on its surface roughness, phase transformation, and biaxial flexural strength (BFS) in pre-sintered and post-sintered stages.	Forty zirconia ceramic discs. Size: 1.2 mm in thickness and 12 mm in diameter.	Pre-sintered and post-sintered zirconia. Bruxzir Shaded (Glidewell, CA, EEUU)	**5Y-TZP**	The pre-sintered treated group and control group showed no monoclinic phase, while the post-sintered group showed higher portions of monoclinic phase. BFS was higher in post-sintered group.	Air abrasion in the pre-sintered stage might be a surface treatment method to produce better surface roughness without subjecting it to early degradation.
2018 (in vitro)	Juntavee et al. [45] (Thailand)	Evaluate the effect of different sintering temperatures and sintered-holding times on flexural strength of translucent monolithic zirconia.	One hundred and thirty-five (135) zirconia specimens were prepared in a bar shape at the dimension of 12 mm width, 25 mm length and 1.8 mm in thickness from Y-TZP.	Zirconia blanks (Y-TZP, VITA YZ HT color^®^, Vita Zahnfabrik, Säckingen, Germany)	**4Y-TZP**	Increasing sintering temperature resulted in higher flexural strength and prolonged sintered-holding time resulted in higher flexural strength	Flexural strength of monolithic Y-TZP was influenced by modification of sintering temperature and duration of sintering time.
2018 (in vitro)	Camposilvan et al. [12] (Brazil)	Analyze the microstructural features, mechanical properties, translucency, and aging behavior of the current generation of zirconia.	Disc-shaped of four different commercial zirconia materials (n = 20).	*Full strength*: Aadva ST (ST) (Aadva GCTech, Leuven, Belgium) *Enhanced translucency:* Aadva EI (EI) (Aadva GCTech, Leuven, Belgium) *High translucency:* Aadva NT (NT) (Aadva GCTech, Leuven, Belgium) *High translucency multi-layered:* Katana UTML (ML) (Kuraray Noritake Dental Inc, Aichi, Japan)	ST: **3Y-TZP** 0.2% Al_2_O_3_ EI: **3Y-TZP** 0.05% Al_2_O_3_ NT: **5Y-TZP** 0.05% Al_2_O_3_ ML: **5Y-TZP** unknown Al₂O_3_	The high amount of cubic phase in 5Y-TZP improves translucency but at the expense of strength and toughness. Hydrothermal degradation takes place in the state-of-the-art 3Y-TZP and is minimal in the third-generation zirconia.	In the current generation of so-called tetragonal zirconia, short aging times have been observed. Glaze acts as a barrier against hydrothermal degradation.
2018 (in vitro)	Yan et al. [39] (EEUU)	Evaluate the load-bearing capacity of monolithic lithium disilicate and novel ultra-translucent zirconia restorative systems of various compositions.	Ten disc-shaped specimens were prepared from three dental zirconia and lithium disilicate (n = 10). Size: 1.0 mm in thickness and 12 mm in diameter.	Dental zirconias. (Luxisse series; Heany Industries, EEUU) Lithium disilicate (IPS e.max CAD; Ivoclar Vivadent, Lichtenstein).	**5Y-TZP** **4Y-TZP** **3Y-TZP**	Load-bearing capacity of LiDi (872 N) is superior to 5Y-PSZ (715 N) when bonded and supported by dentin-like substrate, while 3Y-TZP still holds the highest load-bearing capacity (1195 N). The translucency of 5Y-PSZ approaches that of LiDi, which are superior to both 4Y-PSZ and 3Y-TZP.	When adhesively bonded to and supported by dentin, lithium disilicate exhibits similar load-bearing properties to 4Y-PSZ but much better than 5Y-PSZ
2017 (in vitro)	Schriwer et al. [46] (Norway)	Evaluate whether factors of the production methods or the material composition affect load at fracture, fracture modes, internal fit, or crown margins of monolithic zirconia crowns.	Sixty crowns made of different dental zirconia materials.	Soft-machined (3) Hard-machined (2) Prismatik BruxZir (Glidewell Laboratories) Dental Direkt (Bio ZX2, Dental Direkt GmbH) ZirkonZahn, (Zirconia—Prettau Zirconia) NobelProcera (Nobel Biocare) Denzir Y-TZP, Denzir Mg-PSZ, (Denzir AB)	**5Y-TZP** **4Y-TZP** **3Y-TZP**	Internal fit: statistically significant differences in internal fit at occlusal cement gap. Margin quality: hard-machined had almost flawless margins, less severe margin defects. Load fracture: correlation between the severity of margin defects and load fracture.	Production method and composition affect internal fit, crown margin quality, and the load at fracture of crowns. Hard-machined offers better characteristics.
2016 (in vitro)	Pozzobon et al. [25] (Brazil)	Evaluate the effect of air abrasion.	Eighty blocks.	3Y-TZP ceramic blocks (VITA In-Ceram YZ, Vita Zahnfabrik, Bad Säckingen, Germany).	**3Y-TZP**	Bond strength is affected by particle size factor; 110 µm particles promoted higher bond strength.	The 30 µm and 110 µm silica coating created t-m phase transformation.
2016 (in vitro)	Schatz et al. [29] (Germany)	Evaluate the influence of specimen preparation and test method on flexural strength of monolithic zirconia.	Total of 720 specimens.	Pre-sintered zirconia Ceramill Zolid (Amann Girrbach, Koblach, Austria) Zenostar Zr Translucent (Wieland + Dental, Pforzheim, Germany) DD Bio zx2 (Dental Direkt, Spenge, Germany)	**5Y-TZP** **4Y-TZP** **3Y-TZP**	The different polishing procedures influenced the mean flexural strength independently of which zirconia was tested and which test method was applied. After sintering the wet polished specimen produced significantly higher flexural strength than specimens polished before sintering.	The specimen preparation method significantly impacts the flexural strength; roughness was higher with dry polished specimens.
2016 (in vitro)	Bomicke et al. [30] (Germany)	Compare ultimate fracture load, load at first damage, and fracture pattern for posterior fixed dental prosthesis (FDPs) manufactured from translucent Y-TZP.	Premolar sized FDPs in 4 groups (n = 16)	Y-TZP (Cercon ht medium; DeguDent GmbH). Veneered complete crown retained. Monolithic complete crown retained. Monolithic partial veneer crown retained. Monolithic resin bonded	**5Y-TZP**	No debonding or structural defects detected before fracture loading.	Cracking of veneer occurred. Monolithic 270° partially veneered crown retained, which seemed to debond at forces below 500 N.
2016 (in vitro)	Zhang et al. [8] (Belgium)	Evaluate the optical properties, mechanical properties, and aging stability of Y-TZP with different compositions.	Five different Y-TZP materials were used to evaluate different properties as follows: translucency parameter (TP) (n = 10), bending strength (n = 10), fracture toughness (n = 8), Vickers hardness (n = 10).	Zpex Smile 5Y-TZP (Tosoh, Japan)	**3Y-0.05Al**: 0.05% Al_2_O_3_ **5Y-0.05Al**: 0.05% Al_2_O_3_ Lab made powders: **3Y-0.25Al-0.2La** 0.25% Al_2_O_3_ and 0.2% La_2_O_3_ **3Y-0.1Al-0.2La**: 0.1% Al_2_O_3_ and 0.2% La_2_O_3_	Lowering the alumina content below 0.25% increased the translucency of 3Y-TZP ceramics, but hydrothermal aging stability was reduced. Higher yttria content introduced 50% of cubic zirconia phase and gave rise to the most translucent and aging-resistant; but fracture toughness and strength were sacrificed.	Slight variation in composition resulted in a substantially different translucency, mechanical properties, and hydrothermal stability. Adding 0.2%mol La_2_O_3_ can modify the grain boundary chemistry by means of segregation and provide a promising combination of higher translucency, better aging stability, and similar mechanical properties.
2016 (in vitro)	Pilo et al. [26] (Israel)	Evaluate changes in surface chemistry of Y-TZP frameworks induced by zirconia primer treatments.	Discs of Y-TZP were treated with different primers.	Lava (3M ESPE Seefeld, Germany) *Zirconia primers*: Bisco Z-Prime Plus Bisco, Inc, Schaumburg IL, EEUU) Danville Z-Bond (Danville Materials Inc, S Ramon, CA, EEUU)	**3Y-TZP**	Both primers induced carboxylate salt formation on Y-TZP, promoting chemical adhesion.	Primers produced strongly adsorbed films on Y-TZP, with evidence of carboxylate and phosphate salts formation.

Abbreviations: ***TZP***—tetragonal zirconium dioxide polycrystals; ***FSZ***—fully stabilized zirconia; ***Y-TZP***—yttrium oxide partially stabilized tetragonal zirconium dioxide polycrystals; **3Y-TZP**—3 mol% yttria-stabilized tetragonal zirconia polycrystals; **4Y-TZP**—4 mol% yttria-stabilized tetragonal zirconia polycrystals; **5Y-TZP**—5 mol% yttria-stabilized tetragonal zirconia polycrystals; **BFS**—biaxial flexural strength; **VHN**—Vickers hardness; **ΔE_00_**—CIEDE 2000; **FDPs**—fixed dental prosthesis.

## References

[1] Cokic S.M., Cóndor M., Vleugels J., Van Meerbeek B., Van Oosterwyck H., Inokoshi M., Zhang F. (2022). Mechanical properties-translucency-microestructure relationships in commercial monolayer and multilayer monolithic zirconia ceramics. Dent. Mater..

[2] Zhigachev A.O., Rodaev V.V., Zhigacheva D.V. (2019). The effect of titania doping on structure and mechanical properties of calcia-stabilized zirconia ceramic. J. Mater. Res. Technol..

[3] Vieira Cardoso K., Adabo G.L., Mariscal-Muñoz E., Gutierres Antonio S., Neudenir Arioli Filho J. (2020). Effect of sintering temperature on microstructure, flexural strength, and optical properties of a fully stabilized monolithic zirconia. J. Prosthet. Dent..

[4] Vagkopoulou T., Koutayas S., Koidis P., Strub J.R. (2009). Zirconia in Dentistry: Part 1. Discovering the Nature of an Upcoming Bioceramic. Eur. J. Esthet. Dent..

[5] Stawarczyk B., Keul C., Eichberger M., Figge D., Edelhoff D., Lümkemann N. (2017). Three generations of zirconia: From veneered to monolithic. Part II. Quintessence Int..

[6] Güth J., Stawarczyk B., Edelhoff D., Libermann A. (2019). Zirconia and its novel compositions: What do clinicians need to know?. Quintessence Int..

[7] Zarone F., Di Mauro M., Ausiello P., Ruggiero G., Sorrentino R. (2019). Current status on lithium disilicate and zirconia: A narrative review. BMC Oral Health.

[8] Zhang F., Inokoshi M., Batuk M., Hadermann J., Naert I., Van Meerbeek B., Vleugens J. (2016). Strength, toughness and aging stability of highly-translucent Y-TZP ceramics for dental restorations. Dent. Mater..

[9] Jansen J., Lümkemann N., Letz I., Pfefferle R., Sener B., Stawazcyk B. (2019). Impact of the high-speed sintering on translucency phase content, grain size, and flexural strength of 3Y-TZP and 4Y-TZP zirconia materials. J. Prosthet. Dent..

[10] Stawarczyk B., Keul C., Eichberger M., Figge D., Edelhoff D., Lümkemann N. (2017). Three generations of zirconia: From veneered to monolithic. Part I. Quintessence Int..

[11] Özkurt-Kayahan Z. (2016). Monolithic zirconia: A review of the literature. Biomed. Res..

[12] Camposilvan E., Leone R., Gremillard L., Sorrentino R., Zarone F., Ferrari M., Chevalier J. (2018). Aging resistance, mechanical properties and translucency of different Yttria-stabilized zirconia ceramics for monolithic dental crown applications. Dent. Mater..

[13] Zhang F., Van Meerbeek B., Vleugels J. (2020). Importance of tetragonal phase in high-translucent partially stabilized zirconia for dental restorations. Dent. Mater..

[14] Tang Z., Zhao X., Wang H. (2021). Quantitative analysis on the wear of monolithic zirconia crowns on antagonist teeth. BMC Oral Health.

[15] Moqbel N., Al-Akhali M., Wille S., Kern M. (2020). Influence of Aging on Biaxial Flexural Strength and Hardness of Translucent 3Y-TZP. Materials.

[16] De Araújo-Junior E.N.S., Bérgamo E.T.P., Bastos T.M.C., Benalcázar Jalkh E.B., Lopes A.C.O., Monteiro K.N., Cesar P.F., Tognolo F.C., Migliati R., Tanaka R. (2022). Ultra-translucent zirconia processing and aging effect on microstructural, optical, and mechanical properties. Dent. Mater..

[17] Turgut S. (2020). Optical properties of currently used zirconia-based esthetic restorations fabricated with different techniques. J. Esthet. Restor. Dent..

[18] Sulaiman T.A. (2020). Materials in digital dentistry—A review. J. Esthet. Restor. Dent..

[19] Tovar-Vargas D., Turon-Vinasc M., Anglada M., Jimenez-Pique E. (2020). Enhancement of mechanical properties of ceria-calcia stabilized zirconia by alumina reinforcement. J. Eur. Cer. Soc..

[20] Vardhamana S., Borbaa M., Kaizer M., Kima D., Zhang Y. (2020). Wear behavior and microstructural characterization of translucent multilayer zirconia. Dent. Mater..

[21] Hjerppe J., Steyern P. (2019). Two decades of zirconia as a dental biomaterial—What have we learned?. Tandlægebladet.

[22] Hayashi S., Homma S., Takanashi T., Hirano T., Yoshinari M., Yajima Y. (2019). Wear properties of esthetic dental materials against translucent zirconia. Dent. Mater. J..

[23] Zhang F., Spies B., Vleugels J., Reveron H., Wesemann C., Müller W.D., Van Meerbeek B., Chevalier J. (2019). High-translucent Yttria-stabilized zirconia ceramics are wear-resistant and antagonist-friendly. Dent. Mater..

[24] Skjold A., Schriwer C., Øilo M. (2019). Effect of margin design on fracture load of zirconia crowns. Eur. J. Oral Sci..

[25] Pozzobon J., Missaua T., Druck C.C., Özcan M., Valandro L.F. (2016). Effects of different particle deposition parameters on adhesion of resin cement to zirconium dioxide and phase transformation. J. Adh. Sci. Tech..

[26] Pilo R., Kaitsas V., Zinellis S., Eliades G. (2016). Interaction of zirconia primers with Yttria-stabilized zirconia surfaces. Dent. Mater..

[27] Atoche-Socola K., Arriola-Guillén L., López-Flores A., Garcia I., Huertas-Mogollón G., Mezzomo F., Castelo V. (2021). Microshear bond strength of dual-cure resin cement in zirconia after different cleaning techniques: An in vitro study. J. Adv. Prosthodont..

[28] Kurtulmus-Yilmaz S., Önöral Ö., Aktore H., Ozan O. (2020). Does the application of surface treatments in different sintering stages affect flexural strength and optical properties of zirconia?. J. Esthet. Restor. Dent..

[29] Schatz C., Strickstrock M., Roos M., Edelhoff D., Eichberger M., Zylla I.M., Stawarczyk B. (2016). Influence of Specimen Preparation and Test Methods on the Flexural Strength Results of Monolithic Zirconia Materials. Materials.

[30] Bömicke W., Rues S., Hlavacek V., Rammelsberg P., Schmitter M. (2016). Fracture Behavior of Minimally Invasive, Posterior, and Fixed Dental Prostheses Manufactured from Monolithic Zirconia. J. Esthet. Restor. Dent..

[31] Auzani M.L., Dapieve K.S., Zucuni C.P., Rocha Pereira G.K., Valandro L.F. (2020). Influence of shading technique on mechanical fatigue performance and optical properties of a 4Y-TZP ceramic for monolithic restorations. J. Mech. Behav. Biomed. Mater..

[32] Zucuni C.P., Rocha Pereira G.K., Valandro L.F. (2020). Grinding, polishing and glazing of the occlusal surface do not affect the load-bearing capacity under fatigue and survival rates of bonded monolithic fully-stabilized zirconia simplified restorations. J. Mech. Behav. Biomed. Mater..

[33] Della Bona A., Pecho O., Alessandretti R. (2015). Zirconia as a Dental Biomaterial. Materials.

[34] Tabatabaian F., Khodaei M., Namdari M., Mahshid M. (2016). Effect of cement type on the color attributes of a zirconia ceramic. J. Adv. Prosthodont..

[35] Sui Y., Han L., Jiang Y. (2018). Effect of Ta_2_O_5_ addition on the microstructure and mechanical properties of TiO_2_-added Yttria-stabilized zirconia-toughened alumina (ZTA) composites. Ceramics Int..

[36] Elsayed A., Meyer G., Wille S., Kern M. (2019). Influence of the Yttrium content on the fracture strength of monolithic zirconia crowns after aging. Quintessence Int..

[37] Kolakarnprasert N., Kaizer M., Kim D.K., Zhang Y. (2019). New multi-layered zirconias: Composition, microstructure and translucency. Dent. Mater..

[38] Benalcázar Jalhh E.B., Bergamo E.T.P., Monteiro K.N., Cesar P.F., Genova L.A., Lopes A.C.O., Lisboa Filho P.N., Coelho P.G., Santos C.F.d., Bortolin F. (2020). Aging resistance of an experimental zirconia-toughened alumina composite for large span dental prostheses: Optical and mechanical characterization. J. Mech. Behav. Biomed. Mater..

[39] Yan J., Kaizer M., Zhang Y. (2018). Load-bearing of Lithium Disilicate and Ultra-translucent Zirconias. J. Mech. Behav. Biomed. Mater..

[40] Page M.J., Moher D., Bossuyt P.M., Boutron I., Hoffman T.C., Mulrow C.D., Shamseer L., Tetzlaff J.M., Akl E.A., Brennan S.E. (2021). PRISMA 2020 explanation and elaboration: Updated guidance and exemplars for reporting systematic reviews. BMJ.

[41] Yu N.-K., Mi-Gyoung P. (2019). Effect of different coloring liquids on the flexural strength of multilayered zirconia. J. Adv. Prosthodont..

[42] Kou W., Gabrielsson K., Borhani A., Carlborg M., Thóren M.M. (2019). The effect of artificial aging on high translucent zirconia. Biomat. Investig. Dent..

[43] Elsaka S. (2019). Optical and Mechanical properties of newly developed Monolithic Multilayer Zirconia. J. Prosthodont..

[44] Ebeid K., Wille S., Salah T., Wahsh M., Zohdy M., Kern M. (2018). Evaluation of surface treatments of monolithic zirconia in different sintering stages. J. Prosthodont. Res..

[45] Juntavee N., Attashu S. (2018). Effect of different sintering process on flexural strength of translucency monolithic zirconia. J. Clin. Exp. Dent..

[46] Schriwer C., Skjold A., Gjerdet N., Øilo M. (2017). Monolithic zirconia dental crowns, internal fitting, margin quality, fracture mode and load at fracture. Dent. Mater..

[47] Kontonasaki E., Athanasios E., Rigos A., Ilia C., Istantos T. (2019). Monolithic Zirconia: An Update to Current Knowledge. Optical Properties, Wear, and Clinical Performance. Dent. J..

[48] Mao L., Kaizer M.R., Zhao M., Guo B., Song Y.F., Zhang Y. (2018). Graded Ultra-Translucent Zirconia (5Y-PSZ) for Strength and Functionalities. J. Dent. Res..

[49] Papageorgiou-Kyrana K., Fasoula M., Kontonasaki E. (2020). Translucency of Monolithic Zirconia After Hydrothermal Aging: A Review of In Vitro Studies. J. Prosthodont..

[50] Souza Dantas T., Silveira Rodrigues R.C., Zago Naves L., Lapria Faria A.C., Palma-Dibb R.G., Faria Ribeiro R. (2019). Effects of Surface Treatments on Mechanical Behavior of Sintered and Pre-sintered Yttria-Stabilized Zirconia and Reliability of Crowns and Abutments Processed by CAD-CAM. Int. J. Oral Maxillofac. Implant..

[51] Chena B., Yana Y., Xieb H., Mengc H., Zhang H., Chen C. (2020). Effects of Tribochemical Silica Coating and Alumina-Particle Air Abrasion on 3Y-TZP and 5Y-TZP: Evaluation of Surface Hardness, Roughness, Bonding, and Phase Transformation. J. Adhes. Dent..

[52] Alammar A., Blatz M.B. (2022). The resin bond to high-translucent zirconia—A systematic review. J. Esthet. Restor. Dent..

[53] Baldi A., Comba A., Ferrero G., Italia E., Michelotto Tempesta R., Paolone G., Mazzoni A., Breschi L., Scotti N. (2022). External gap progression after cyclic fatigue of adhesive overlays and crowns made with high translucency zirconia or lithium silicate. J. Esthet. Restor. Dent..

[54] Tang Z., Zhao X., Wang H., Liu B. (2019). Clinical evaluation of monolithic zirconia crowns for posterior teeth restorations. Medicine.

